# Combined Effects of Unhealthy Lifestyle Behaviors on Metabolic Syndrome among Postmenopausal Women

**DOI:** 10.3390/healthcare9070848

**Published:** 2021-07-05

**Authors:** Jin-Suk Ra, Hyesun Kim

**Affiliations:** 1 College of Nursing, National University, Daejeon 35015, Korea; jinsukra@cnu.ac.kr; 2 Department of Nursing, National University Hospital, Chungbuk 28644, Korea

**Keywords:** lifestyle, metabolic syndrome, postmenopause, sedentary behavior

## Abstract

This study aimed to identify the combined effects of unhealthy lifestyle behaviors, including diet, sedentary behavior, and physical activity on metabolic syndrome (MS) and components of MS among postmenopausal women. Secondary data analysis was conducted using the Korean National Health and Nutrition Examination Survey (2014–2018) with a cross-sectional study design. Logistic regression analysis was conducted with data from 6114 Korean postmenopausal women. While no significant effects of unhealthy lifestyle behaviors, either individually or as a combination, were found for MS, prolonged sedentary behavior without poor dietary behavior and insufficient physical activity was associated with increased likelihood of abdominal obesity (adjusted odds ratio [AOR]: 1.59, 95% confidence interval [CI]: 1.10–2.29) and impaired fasting glucose (AOR: 1.54, 95% CI: 1.13–2.10). The combination of poor dietary behavior and prolonged sedentary behaviors was also associated with increased likelihood of abdominal obesity (AOR: 1.48, 95% CI: 1.10–2.00) and impaired fasting glucose (AOR: 1.49, 95% CI: 1.14–1.96). In addition, prolonged sedentary behavior and insufficient physical activity together were associated with increased likelihood of abdominal obesity (AOR: 2.81, 95% CI: 1.90–4.20) and impaired fasting glucose (AOR: 1.59, 95% CI: 1.13–2.24). Finally, combining poor dietary behavior, prolonged sedentary behavior, and insufficient physical activity was also associated with increased likelihood of abdominal obesity (AOR: 2.05, 95% CI: 1.50–2.80) and impaired fasting glucose (AOR: 1.71, 95% CI: 1.32–2.23). Strategies for replacing sedentary behavior of postmenopausal women with activities are warranted for prevention of abdominal obesity and impaired fasting glucose.

## 1. Introduction

Metabolic syndrome (MS) is a cluster of cardiovascular and metabolic risk factors, which includes elevated blood pressure, abdominal (visceral) obesity, altered lipid metabolism, and impaired glucose levels. The development of MS has been associated with increased age and unhealthy lifestyle behaviors, such as poor dietary behavior (e.g., excessive intake of calories), prolonged sedentary behavior, and insufficient physical activity [1]. According to the national data of the United States of America, between 2011 and 2016, 34.7%–50.4% of adults aged 40 to 60 years had MS; in contrast, the prevalence of MS was 16.2%–21.3% in those aged 20 to 39 years [2]. In Korea, 30%–40% of adults aged over 40 years had MS between 2013 and 2015 [3]. Thus, this syndrome may be a significant health problem among middle-aged and older individuals. 

Previous studies have reported that the prevalence of MS was greater among middle-aged and older women than men of the same age [4,5]. Moreover, an increased prevalence of MS may be associated with menopause among middle-aged and older women [5,6]. Menopause promotes abdominal obesity with changes in body fat distribution, which is associated with an increased risk of insulin resistance, dyslipidemia, and hypertension [6,7,8]. In the same vein, a previous study reported that postmenopausal women had a greater risk of type 2 diabetes mellitus (T2DM), low high-density lipoprotein (HDL) cholesterol, high low-density lipoprotein (LDL) cholesterol levels, and hypertension [9,10]. Hence, postmenopausal women are considered high-risk individuals for MS and its complications, such as cardiovascular diseases (CVDs).

An increased risk of MS is associated with unhealthy lifestyle behaviors, including poor dietary behaviors, prolonged sedentary behaviors, and insufficient physical activity [11,12,13]. Since dietary intake and physical activity are major variables in our daily lives, these are also significant determinants of MS and components of MS such as waist circumference, blood pressure, HDL cholesterol, triglyceride, and fasting blood sugar [12,13,14,15]. According to a study by Myers et al. [16], sufficiently increased physical activity was effective for prevention and management of MS and components of MS, including abdominal obesity and insulin resistance. Similarly, a high calorie diet, prolonged sedentary behaviors, and insufficient physical activity might be independently associated with an increased prevalence of MS in women aged 30 years and above, with sex differences on the impact of lifestyle factors [11,12,13].

However, individuals tend to combine lifestyle factors, with a cluster of specific patterns of lifestyle behaviors [17]. Furthermore, the combined effects of lifestyle behaviors may be different from the independent effect of each lifestyle behavior [18]. The combined effect of lifestyle behaviors on health may be neutralized or reinforced owing to a synergistic effect between the behaviors [19]. Previous studies have shown that combining the effects of sufficient physical activity and healthy dietary habits was significantly better for relieving the risk of MS (e.g., abdominal obesity, high fasting blood sugar) than their individual effects [20]. On the other hand, combining unhealthy lifestyle behaviors was more associated with increased risk of MS than with the sum of the risk of MS associated with each of the individual unhealthy lifestyle behaviors (e.g., poor dietary behaviors, insufficient physical activity) [21]. Postmenopausal middle-aged women showed decreased physical activity with increasing age [22], and their energy intake was higher than that of the other age groups of women and men of the same age [23]. In addition, a modernized lifestyle, prolonged sedentary behaviors and decreased sufficient physical activity resulted in greater risk of MS [24]. Thus, it is necessary to evaluate the potential combined effects of diet, sedentary behavior, and physical activity on MS and components of MS in postmenopausal women. However, we found that there are limited reports on this association in postmenopausal women.

The biopsychosocial model proposed by Hoffman and Driscoll [25] may provide a framework for considering the effects of various covariates for MS, including biomedical factors (e.g., biological process and genetics), biosocial factors (e.g., sex, race/ethnicity, environmental characteristics such as education, socioeconomic status), and psychosocial factors, including emotional status and health-related behaviors (e.g., mood and behaviors) [26]. Based on a literature review following this model, the biomedical factors including age, body mass index (BMI), and family history of hypertension, type 2 diabetes mellitus (T2DM), CVDs, and dyslipidemia, were considered as covariates [5,27,28,29]. Family socioeconomic status and educational level were included as biosocial factors [30,31]. Finally, psychosocial factors such as stress, depression, current or past smoking history, current binge alcohol consumption, and sleep duration were included [29,31]. Thus, the combined effects of unhealthy lifestyle behaviors (poor dietary behavior, prolonged sedentary behavior, and insufficient physical activity) on MS and components of MS among postmenopausal women were identified by controlling the covariates of biomedical, biosocial, and psychosocial factors.

## 2. Materials and Methods

### 2.1. Design and Participants

In this cross-sectional study, the secondary data of the 2014–2018 Korean National Health and Nutrition Examination Survey (KNHNES) were analyzed. In most Korean women, menopause occurs after the age of 40 [32]. Among the 39,199 women who participated in the KNHNES, 12,764 women aged >40 years were selected. In addition, among the selected 12,764 women, 6650 women, including 2823 who were premenopausal and 3827 who did not complete a questionnaire assessing health and nutritional status, were excluded. Finally, 6114 women were eligible for the study for data analysis. 

### 2.2. Measurements

#### 2.2.1. Outcome Variable

##### Metabolic Syndrome

MS was defined in accordance with the diagnostic criteria of the American Heart Association and the National Heart, Lung, and Blood Institute (AHA/NHLBI) [1]. For abdominal obesity in Korean women, waist circumference was evaluated according to criteria suggested by the Korean Society for the Study of Obesity, which reflected optimal waist circumference predicting MS of Korean women [33]. Among the five components for the diagnosis of MS, individuals with three and more components were considered to have MS. The diagnostic criteria were as follows: (i) abdominal obesity: ≥85 cm; (ii) elevated blood pressure: ≥130/85 mmHg or on anti-hypertensive medications; (iii) low HDL cholesterol levels: <50 mg/dL or on anti-dyslipidemic medications; (iv) high triglycerides levels: ≥150 mg/dL or on anti-dyslipidemic medications; and (v) impaired fasting glucose levels: ≥100 mg/dL or on anti-diabetic medications.

#### 2.2.2. Unhealthy Lifestyle Behaviors

##### Dietary Behavior

Dietary behavior associated with MS was evaluated using the following criteria [21]: (i) >125% of the estimated daily total calorie requirement; (ii) >75% of the recommended daily total intake calories from carbohydrates; (iii) >30% of recommended daily total intake calories from fat; (iv) >130% of the recommended mean sodium intake per day for Koreans. Individuals with more than one dietary criterion among the four were considered to have poor dietary behavior predominantly associated with MS.

##### Sedentary Behavior

Sedentary behavior was measured using a single question: “How many hours do you usually sit or lie down a day?” Responses were categorized into <8 h a day and ≥8 h a day; sedentary time of more than 8 h a day was considered as prolonged sedentary behavior according to the guidelines of the United Kingdom Chief Medical Officers [34]. 

##### Physical Activity

Physical activity was quantified using the Metabolic Equivalent of Task (MET)-minutes of Global Physical Activity Questionnaire (GPAQ) analysis guide (version 2.0) [35]. Physical activity included activities during work, transport, and recreation. This was computed as the sum of transport-related walking or cycling, as well as moderate and vigorous activities in a usual week, measured as metabolic equivalents. Physical activity based on total MET-min/week was categorized as sufficient physical activity (≥600 MET-min/week) and insufficient physical activity (<600 MET-min/week) in accordance with the WHO guideline [35].

#### 2.2.3. Covariates

##### Biomedical Factors

Age was divided into two categories (40–64 years and ≥65 years). BMI was computed from objectively measured weight and height, and it was divided into two categories (underweight and normal weight (<23 kg/m^2^); overweight and obese (≥23 kg/m^2^)) [36]. Family history of hypertension, T2DM, CVDs, dyslipidemia was evaluated using questions on the history of hypertension, T2DM, CVDs, and dyslipidemia to direct family members. The response was either “yes” or “no”. 

##### Biosocial Factors

The highest level of educational attainment was considered the participant’s level of education. The responses were categorized into four groups (lower than elementary school education, graduation from middle school, graduation from high school, college education or above). The socioeconomic status of the family was determined using the level of family income. The responses were categorized into three groups (high, middle, or low). 

##### Psychosocial Factors

Stress was measured using a single question: “How much stress do you feel in your daily life?”. The respondents were categorized as being under severe stress or a little stress or not at all under stress. Depression was evaluated using a single question: “Do you have depression diagnosed by a psychiatrist?”. Current or past smoking history was assessed using a single question on current or past smoking experiences. Responses were categorized into “yes” or “no”. Current binge alcohol consumption was screened with the Alcohol Use Disorders Identification Test Alcohol Consumption Questions (AUDIT-C) that include three questions (amount and frequency of alcohol consumption and frequency of heavy drinking). The total score ranged from 0 to 12 points, with 0 to 4 points being the response rate of each item. More than six points was considered as binge alcohol consumption, based on the criteria proposed by Woo and his colleagues [37]. Sleep duration was divided into two groups, less than 7 h and more than 7 h according to the results of the meta-analysis [38].

### 2.3. Data Analysis

Data analyses were conducted using complex simple analysis procedures of SPSS version 24.0 (IBM, Armonk, NY, USA) using the guide for data analysis proposed by the Korean National Health and Nutrition Examination Survey. The characteristics of MS, dietary behavior, sedentary behavior, physical activity, and covariates (biomedical, biosocial, and psychosocial factors) were described using frequencies and percentages. Logistic regression analysis was conducted to adjust for covariates, in order to identify the combined effects of unhealthy lifestyle behaviors (poor dietary behavior, prolonged sedentary behavior, and insufficient physical activity) on MS and components of MS among postmenopausal women. 

## 3. Results

### 3.1. Characteristics of Metabolic Syndrome, Unhealthy Lifestyle, Covariates among Participants

In total, 45.7% of the participants had MS. The prevalence of the five individual MS components is presented in Table 1. Among the total participants, 39.5% had at least one of the three unhealthy lifestyle behaviors associated with MS, namely, poor dietary behavior, prolonged sedentary behavior, or insufficient physical activity; 36.8% of the participants had two of the three unhealthy lifestyle behaviors associated with MS; and 14.5% of the participants had all three unhealthy lifestyle behaviors. Table 1 shows the prevalence of the covariates, including biomedical, biosocial, and psychosocial factors.

### 3.2. Combined Effects of Unhealthy Lifestyle Behaviors on Metabolic Syndrome

No significant effects of unhealthy lifestyle behaviors, either individually or as a combination of two or more, were found for MS, after adjusting for covariates (Table 2). However, prolonged sedentary behavior, but not poor dietary behavior or insufficient physical activity, was associated with an increased likelihood of abdominal obesity (adjusted odds ratio [AOR]: 1.59, 95% confidence interval [CI]: 1.10–2.29) and impaired fasting glucose levels (AOR: 1.54, 95% CI: 1.13–2.10). A combination of poor dietary behavior and prolonged sedentary behavior was associated with an increased likelihood of abdominal obesity (AOR: 1.48, 95% CI: 1.10–2.00) and impaired fasting glucose levels (AOR: 1.49, 95% CI: 1.14–1.96). Likewise, a combination of prolonged sedentary behavior and insufficient physical activity was associated with an increased likelihood of abdominal obesity (AOR: 2.81, 95% CI: 1.90–4.20) and impaired fasting glucose levels (AOR: 1.59, 95% CI: 1.13–2.24). Furthermore, a combination of poor dietary behavior, prolonged sedentary behavior, and insufficient physical activity also increased the likelihood of abdominal obesity (AOR: 2.05, 95% CI: 1.50–2.80) and impaired fasting glucose levels (AOR: 1.71, 95% CI: 1.32–2.23).

## 4. Discussion

This study identified the combined effects of poor dietary behavior, prolonged sedentary behavior, and insufficient physical activity on MS, and components of MS among postmenopausal women. According to the results of the study, postmenopausal women with prolonged sedentary behavior, not including poor dietary behavior and insufficient physical activity, had an increased likelihood of abdominal obesity and impaired fasting glucose levels. Moreover, abdominal obesity and impaired fasting glucose levels were prevalent among postmenopausal women with prolonged sedentary behavior, combined with poor dietary behavior and/or insufficient physical activity. However, despite having healthy dietary patterns and/or doing sufficient physical activity, postmenopausal women with prolonged sedentary behavior might be at risk for abdominal obesity and impaired fasting glucose levels. Thus, prolonged sedentary behaviors might be primarily significant factors associated with metabolic abnormalities of abdominal obesity and impaired fasting glucose with combined poor dietary behaviors and/or insufficient physical activity.

During the normal aging process, postmenopausal women may be at an increased risk of abdominal obesity owing to estrogen deficiency due to decreased ovarian function. During the premenopausal period, sufficient estrogen levels activate lipoproteins (leading to accumulation of fat as energy stores for reproduction) in the femoral adipose tissue rather than in the abdominal adipocytes [39]. Thus, body fat usually tends to accumulate in the femoral region, rather than in the abdomen among premenopausal women [39]. Meanwhile, postmenopausal women with estrogen deficiency may experience a higher accumulation of visceral fat in the abdomen and a lower accumulation of subcutaneous fat in the femoral region [9,40], than those without estrogen deficiency. 

Furthermore, postmenopausal women tend to have increased sedentary behavior due to decreased muscle strength and endurance for physical activity, with aging [41]. Prolonged sedentary behavior may lead to decreased energy expenditure due to a lack of physical activity [15]. Moreover, a sedentary behavior, such as working on the computer and TV watching may be associated with an increased energy intake as it encourages snacking [42]. Thus, in postmenopausal women, due to a decreased basal metabolic rate and advancing age, prolonged sedentary behavior leads to a positive energy balance by decreasing energy expenditure and increasing energy intake. Sedentary behavior includes waking activities requiring a resting level of energy expenditure (1.0~1.5 basal metabolic rate), such as sitting (e.g., TV watching) and reclining posture [43]. According to previous studies that objectively measured sedentary behaviors, prolonged sedentary behavior was associated with a greater waist circumference [44,45]. In a systematic review, prolonged sedentary time was associated with a greater waist circumference in those aged 60 years or more [46]. Bankoski and colleagues [30] reported that regardless of the physical activity, the association between objectively measured sedentary time and waist circumference was significantly positive with controlled covariates (sex, age, ethnicity, BMI, smoking, and alcohol consumption). Hence, a decrease in sedentary behavior was recommended for alleviating the risk of abdominal obesity, which is a component of MS [30]. Based on these previous results, an increased risk of abdominal obesity among postmenopausal women may be associated with the additional deleterious effects of extended sedentary behavior and the normal aging process after menopause. In addition, minimizing sedentary behavior may be the primary intervention needed to prevent abdominal obesity among postmenopausal women.

Furthermore, the increased body fat and visceral fat of postmenopausal women with normal aging results in an altered expression of adiponectin, which is involved in the regulation of insulin sensitivity, resulting in increased insulin resistance [47]. Furthermore, increased insulin resistance with menopause may be associated with a predilection to sedentary behavior with advancing age [48]. Similarly, self-reported sedentary time was associated with insulin resistance among older adults without T2DM [49]. A previous study involving middle-aged women in The Netherlands found that women with impaired glucose tolerance and T2DM reported an increased sedentary time of up to 26 min per day when compared to women with normal glucose levels, independent of physical activity [50]. Furthermore, as a major leisure-time sedentary behavior, TV watching time was positively associated with the prevalence of T2DM in women, independent of the physical activity [51]. Similarly, among women without diagnosed diabetes, a dose–response relationship was reported between TV watching time and fasting and 2-h serum glucose levels, after controlling for physical activity and waist circumference [52]. Dunstan et al. [52] proposed that sedentary behavior, including TV watching, is a significant lifestyle behavior that disrupts normal metabolic function, independent of physical activity. Thus, along with healthy dietary behavior and sufficient physical activity, prolonged sedentary behavior needs to be reduced for the prevention of insulin resistance, which is associated with impaired glucose tolerance and T2DM. For individuals with more than 9 h of sedentary time per day, regularly performed light activity (walking for 30 min) which interrupts sedentary behaviors was effective in lowering glucose levels after meals [53]. In addition, Yates and colleagues [54] proposed that replacing 30 min of sedentary behavior with light movement increased insulin sensitivity by 5% among individuals with a high risk of T2DM. Thus, sedentary behavior needs to be replaced with at least light physical activity, in order to maintain normal glucose levels in postmenopausal women. 

In our results, the unhealthy lifestyle behaviors were not associated factors on MS among postmenopausal women. According to a previous study of Asians aged above 60 years, poor dietary behaviors such as excessive carbohydrate intake was not associated with MS, and the association between physical activity and MS was inconsistent according to sex [14]. Thus, association between the unhealthy lifestyle behaviors and MS might be influenced by the biological background of individuals, such as sex and race/ethnicity. In addition, association between the unhealthy lifestyle behaviors and MS might be controlled by strong covariates, such as age and BMI. According to Serrano-Sánchez et al. [55], age and BMI had an explanation power of 45.1% and 34.3%, respectively, while that of physical activity was 10%. In addition, a combination of regular physical activity and healthy diet in managing BMI was primarily recommended to decrease the risk of MS [56].

With a large sample size, this study verified the important effects of prolonged sedentary behavior including abdominal obesity and impaired fasting glucose levels, in postmenopausal women. However, this study had several limitations. First, this study used a cross-sectional study design, which does not verify the causal relationship between independent and outcome variables; thus, longitudinal studies are required. Second, the unhealthy lifestyle behaviors were assessed using a self-reported questionnaire; thus, subjective evaluation can be under- or overestimated following social desirability, in comparison to objective measurements. Hence, associations between objectively measured unhealthy lifestyle behaviors and MS need to be verified in future studies. Third, the study included only Korean postmenopausal women. However, patterns of unhealthy lifestyle behaviors associated with MS would differ according to the socio-cultural background of the participants. Thus, a similar study with samples from various cultures is warranted. Fourth, for a combination of unhealthy lifestyle behaviors associated with MS, poor dietary behavior, prolonged sedentary behavior, and insufficient physical activity were selected. Hence, further studies combining other unhealthy lifestyle behaviors associated with MS, such as smoking and alcohol consumption are necessary. Fifth, waist circumference was used as a surrogate marker for abdominal (visceral) obesity; however, since it is an indirect measurement, it is necessary to evaluate this aspect using a more objective and direct measurement (e.g., ultrasonography) in future studies. Sixth, although there are various diagnostic criteria for MS (e.g., International Diabetes Federation, National Cholesterol Education Program-Adult Treatment Panel III, AHA/NHLBI), we used the AHA/NHLBI criteria for diagnosis of MS, which are the most commonly used criteria worldwide. Thus, when different diagnostic criteria are used for MS, the results (e.g., prevalence of MS) may differ from our results, so care must be taken in the interpretation of our results.

## 5. Conclusions

Estrogen is a critical hormonal regulator involved in the development and deposition of adipose tissue. Deficiency of estrogen due to menopause among women induces greater visceral fat, which is associated with abnormal glucose metabolism (e.g., increased insulin resistance and glucose production in the liver and muscle, decreased insulin secretion in the pancreas) [57]. Thus, postmenopausal women might experience abdominal obesity and hyperglycemia. According to the results of this study, prolonged sedentary behavior has a more important role in abdominal obesity and impaired fasting glucose in postmenopausal women than poor dietary behavior and insufficient physical activity. Thus, in addition to estrogen replacement, interventions for decreasing sedentary behavior should be primarily recommended to prevent abdominal obesity and hyperglycemia among postmenopausal women. Healthcare providers, therefore, should develop strategies for replacing sedentary behavior with activities that involve sufficient movements.

## Figures and Tables

**Table 1:** Characteristics of Metabolic Syndrome, Unhealthy Lifestyle Behaviors, and Covariates among Participants (*N* = 6114).

Variables		Categories	*n* (%)
Metabolic syndrome		Yes	2939 (45.7)
		No	3175 (54.3)
Components of metabolic syndrome			
	Abdominal obesity	Yes	2191 (34.0)
		No	3923 (66.0)
	Elevated blood pressure	Yes	3429 (52.6)
		No	2685 (47.4)
	Low high density lipoprotein cholesterol	Yes	3632 (57.4)
		No	2482 (42.6)
	High triglycerides	Yes	2740 (43.3)
		No	3374 (56.7)
	Impaired fasting glucose levels	Yes	2657 (42.7)
		No	3457 (57.3)
Unhealthy lifestyle behaviors			
None			534 (9.2)
One unhealthy lifestyle behavior			
	Poor dietary behaviors		1561 (26.8)
	Prolonged sedentary behaviors		468 (8.1)
	Insufficient physical activity		281 (4.6)
Combining two unhealthy lifestyle behaviors			
	Poor dietary behaviors and prolonged sedentary behaviors		1105 (18.4)
	Poor dietary behaviors and insufficient physical activity		901 (13.4)
	Prolonged sedentary behaviors and insufficient physical activity		319 (5.0)
Combining three unhealthy lifestyle behaviors			
	Poor dietary behaviors, prolonged sedentary behaviors, and insufficient physical activity		945 (14.5)
Covariates			
Biomedical factors			
	Age (years)	40–64	3488 (63.3)
		≥65	2626 (36.7)
	Body mass index	Underweight or normal weight	2304 (39.7)
		Overweight or obese	3810 (60.3)
	Family history of hypertension, type 2 diabetes mellitus, cerebro-cardiovascular disease, dyslipidemia	Yes	3993 (66.2)
		No	2121 (33.8)
Biosocial factors			
	Education level	Lower than elementary school education	2664 (38.1)
		Graduation of middle school	1071 (17.9)
		Graduation of high school	1559 (28.8)
		College education or above	820 (15.2)
	Socioeconomic status of family	High	1396 (26.1)
		Middle	2964 (48.8)
		Low	1754 (25.1)
Psychosocial factors			
	Stress	Severe	1418 (23.8)
		A little or not at all	4696 (76.2)
	Depression	Yes	519 (8.3)
		No	5595 (91.7)
	Current or past smoking history	Yes	372 (6.4)
		No	5742 (93.6)
	Current binge alcohol consumption	Yes	365 (7.1)
		No	5749 (92.9)
	Sleep duration (hours)	<7	2697 (45.7)
		≥7	3417 (54.3)

Note: Values are presented as underweighted number (weighted percentage).

**Table 2:** Combined Effects of Unhealthy Lifestyle Behaviors on Metabolic Syndrome among Postmenopausal Women (*N* = 6114).

Unhealthy Lifestyle Behaviors		Metabolic Syndrome	Abdominal Obesity	Elevated Blood Pressure	Low high Density Lipoprotein Cholesterol	High Triglyceride	Impaired Fasting Glucose Levels
		Adjusted odds ratio (95% Confidence Interval)					
None (Reference)		1.00	1.00	1.00	1.00	1.00	1.00
One unhealthy lifestyle behavior	Poor dietary behaviors	0.89 (0.67–1.18)	1.23 (0.90–1.67)	0.83 (0.65–1.06)	0.87 (0.68–1.10)	0.81 (0.63–1.04)	1.10 (0.85–1.42)
	Prolonged sedentary behaviors	1.34 (0.95–1.87)	1.59 (1.10–2.29) *	0.94 (0.68–1.29)	1.01 (0.75–1.36)	1.02 (0.75–1.37)	1.54 (1.13–2.10) *
	Insufficient physical activity	0.83 (0.57–1.22)	1.08 (0.72–1.62)	0.94 (0.63–1.39)	0.73 (0.50–1.06)	0.80 (0.57–1.12)	1.04 (0.73–1.48)
Combining two unhealthy lifestyle behaviors	Poor dietary behaviors and prolonged sedentary behaviors	1.00 (0.75–1.33)	1.48 (1.10–2.00) *	1.02 (0.79–1.31)	1.08 (0.84–1.40)	0.94 (0.73–1.21)	1.49 (1.14–1.96) *
	Poor dietary behaviors and insufficient physical activity	1.11 (0.81–1.51)	1.21 (0.89–1.64)	1.03 (0.78–1.37)	1.00 (0.76–1.31)	0.92 (0.70–1.20)	1.27 (0.95–1.69)
	Prolonged sedentary behaviors and insufficient physical activity	1.46 (0.99–2.14)	2.81 (1.90–4.20) *	1.2 9(0.90–1.84)	1.01 (0.71–1.45)	0.99 (0.70–1.41)	1.59 (1.13–2.24) *
Combining all three unhealthy lifestyle behaviors	Poor dietary behaviors, prolonged sedentary behaviors, and insufficient physical activity	1.24 (0.91–1.69)	2.05 (1.50–2.80) *	0.98 (0.74–1.30)	1.22 (0.93–1.62)	0.98 (0.75–1.30)	1.71 (1.32–2.23) *

Note 1: Adjusted odds ratio = Adjusted for age, body mass index, family history, education level, economic status, stress, depression, smoking habits, binge alcohol consumption, and sleep duration. Note 2: * = *p* < 0.05. Note 3: Metabolic syndrome = presence of 3 or more of the following: (1) abdominal obesity (waist circumference ≥85 cm), (2) elevated blood pressure: ≥130/85 mmHg or on anti-hypertensive medications, (3) low HDL cholesterol: <50 mg/dL or on anti-dyslipidemic medications, (4) high triglyceride: ≥150 mg/dL or on anti-dyslipidemic medications, (5) impaired fasting glucose levels: ≥100 mg/dL or on anti-diabetic medications.

## Data Availability

Data were obtained from KDCA and are available from https://knhanes.kdca.go.kr/knhanes/main.do (accessed on 3 September 2020).
